# Rag GTPases control lysosomal acidification by regulating v-ATPase assembly in *Drosophila*

**DOI:** 10.1016/j.jbc.2025.110400

**Published:** 2025-06-19

**Authors:** Ying Zhou, Xiaodie Yang, Wenyu Xu, Sulin Shen, Weikang Fan, Guoqiang Meng, Yang Cheng, Yingying Lu, Youheng Wei

**Affiliations:** 1 College of Bioscience and Biotechnology, Yangzhou University, Yangzhou, China; 2 Joint International Research Laboratory of Agriculture and Agri-Product Safety, the Ministry of Education of China, Yangzhou University, Yangzhou, China; 3 Institute of Reproduction and Metabolism, Yangzhou University, Yangzhou, China

**Keywords:** *Drosophila melanogaster*, Rag GTPases, chaperonin-containing tailless complex polypeptide 1, autophagy, v-ATPase assembly

## Abstract

The Rag GTPases play an important role in sensing amino acids and activating the target of rapamycin complex 1, a master regulator of cell metabolism. Previously, we have shown that GDP-bound RagA stimulates lysosome acidification and autophagic degradation, which are essential for young egg chamber survival under starvation in *Drosophila*. However, the underlying mechanism is unclear. Here, we demonstrate that the GDP-bound RagA breaks the physical interaction between cytosolic chaperonin–containing tailless complex polypeptide 1 (CCT) and vacuolar H+-ATPase (v-ATPase) subunit V1, and thus promotes the assembly of active v-ATPase and increases the lysosomal acidification. Consistently, knockdown of CCT complex components rescued the accumulation of defective autolysosomes in *RagA RNAi*. Moreover, the knockdown of Lamtor4, a component of lysosomal adaptor and MAPK and mTOR activator (LAMTOR) that anchors Rag GTPases to the lysosome, resulted in autolysosome accumulation, suggesting that Rag GTPases regulate lysosomal acidification depend on their lysosomal localization. Knockdown of the CCT complex components attenuated the autophagic defects in *Lamtor 4 RNAi*. Our work highlights the interaction between CCT and v-ATPase in regulating lysosomal acidification.

The target of rapamycin complex 1 (TORC1) is a master regulator of cell metabolism that controls cell growth and autophagy. Its activity is mainly controlled by two Ras-related small GTPases, Rag and Rheb (1). Rag GTPases function as heterodimers composed of RagA/B and RagC/D (2). When RagA/B binding GTP and RagC/D binding GDP, Rag GTPases adopt an active conformation that recruits TORC1 to the lysosomal surface for activation (3, 4, 5). The localization of Rag GTPases to the lysosomal membrane is mediated by the late endosomal/lysosomal adaptor and MAPK and mTOR activator (LAMTOR/Ragulator) complex, a pentameric assembly of Lamtor1-5, which exhibits guanine nucleotide exchange factor (GEF) activity toward RagA (6, 7, 8). All five components of the LAMTOR complex are essential for Rag GTPases localized on lysosomes (9, 10). Lamtor1, with the myristoylation and palmitoylation sites, is critical for anchoring the complex to the lysosomal membrane (7, 11). The interactions between Lamtor2-Lamtor3 and Lamtor4-Lamtor5 are essential for the stability of the LAMTOR complex. Depletion of any LAMTOR complex component disrupts the lysosomal localization of Rag GTPases (7, 9, 12). Additionally, Lamtor1 and Lamtor2 interact with vacuolar H^+^-ATPase (v-ATPase) and Rag GTPases, respectively, serving as a functional link between these two complexes (13, 14).

Autophagy is an evolutionarily conserved cellular recycling process. The autophagosome delivers its cargo to fuse with the lysosome and forms an autolysosome, where cargo degradation occurs (15). The lysosomes have an acidic lumen filled with acid hydrolases, which degrade substrates to regenerate nutrients. Hence, the lysosome lumen with a low pH value is essential for cellular homeostasis (16). The v-ATPase is a multisubunit enzyme composed of two domains, V1 and V0 (17). The V1 domain catalyzes ATP hydrolysis in cytoplasm and the V0 domain is embedded in the membrane for proton transfer (18, 19). The assembled state v-ATPase transports protons across the lysosomal membrane into the lysosomal to maintain the acidic pH of the lysosomal lumen (17, 19, 20). When TORC1 is activated, the v-ATPase V0 domain localizes to the lysosome membrane, while the V1 domain interacts with the cytosolic chaperonin–containing tailless complex polypeptide 1 (CCT) in the cytosol (21). TORC1 inactivation triggers the v-ATPase V0 and V1 domains to assemble at the lysosome membrane and activate autophagy degradation (22).

The CCT complex is a double-ring structure, with each ring consisting of eight paralogous subunits (CCT1-8) (23, 24). The CCT complex interacts with protein cofactors to fold specific proteins, playing a crucial role in maintaining cellular protein homeostasis. Moreover, the CCT complex is associated with cell proliferation, cell viability, cell cycle arrest, and cellular apoptosis (25, 26). Several studies reveal that the CCT complex interacts with TOR signaling components to regulate *Drosophila* autophagy, cell division, and organ development (25, 27, 28).

Previously, we have reported that depletion of RagA leads to defects in autolysosomal acidification and degradation. GDP-bound RagA, but not GTP-bound RagA, rescues these defects in *RagA RNAi* (29). Here, we show that v-ATPase assembly deficiency underlies autophagic degradation defects in *RagA RNAi* ovaries. We find that depletion of the CCT complex attenuates autolysosome acidification and degradation defects in *RagA RNAi* ovaries. Furthermore, GDP-bound RagA disrupts the interaction between the CCT complex and the v-ATPase V1 subunit, promoting v-ATPase assembly and lysosomal acidification. Additionally, the lysosomal localization of Rag GTPases, anchored by the LAMTOR complex, is essential for autolysosome acidification and degradation. Our work suggests that GDP-bound RagA associates with the CCT complex to regulate v-ATPase assembly, thereby influencing autolysosome acidification and degradation.

## Results

### Rag GTPases associate with the CCT complex

**Figure 1:**
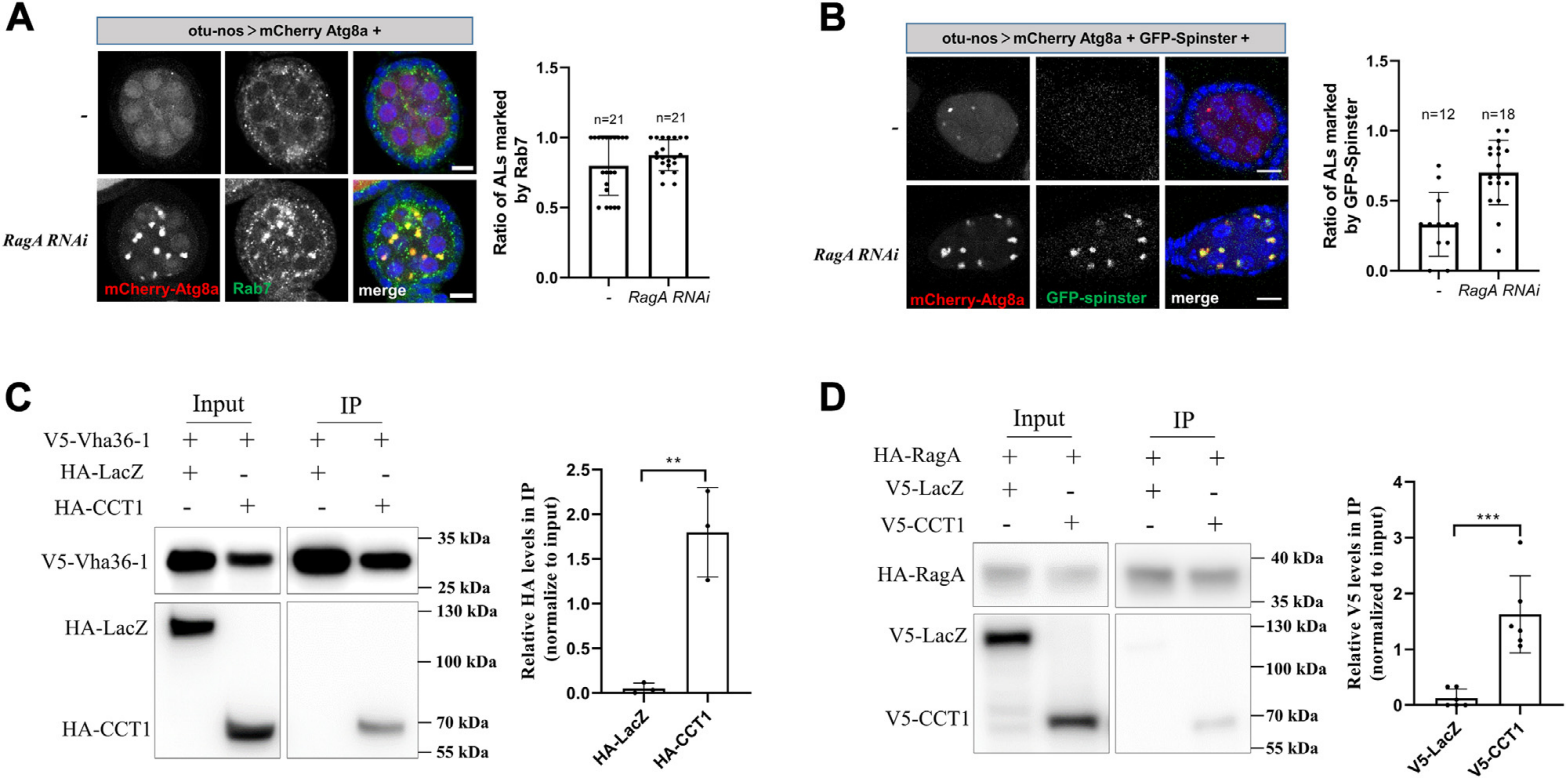
**Rag GTPases associate with CCT complex**. *A*, the ovaries were dissected and stained with anti-Rab7 antibody and DAPI. *Right*: quantitative analyses of the autolysosomes (ALs, marked by Atg8a-mCherry) positive for Rab7 in the egg chambers. *B*, the ovaries were dissected and stained with DAPI. *Right*: quantitative analyses of the autolysosomes (ALs, marked by Atg8a-mCherry) positive for GFP-spinster in the egg chambers. *C*, S2 cells were cotransfected with V5-tagged Vha36-1 and HA-tagged CCT1 or LacZ (control) plasmids. Cells were lysed and immunoprecipitated using beads coated with anti-V5 antibodies. Cell lysates (input) and immunoprecipitates (IP) were detected by Western blot. The experiments were repeated three times, and representative data are shown. *Right*: quantitative analyses of Co-IP of three independent experiments. *D*, S2 cells were cotransfected with HA-tagged RagA and V5-tagged CCT1 or LacZ (control) plasmids. Cells were lysed and immunoprecipitated using beads coated with anti-HA antibodies. Cell lysates (input) and immunoprecipitates (IP) were detected by Western blot. The experiments were repeated six times, and representative data are shown. *Right*: quantitative analyses of co-IP of six independent experiments. For all graphs, mean and SEM with all data points are shown. n indicates the number of egg chambers. ∗∗*p* < 0.01; ∗∗∗*p* < 0.001. The scale bar represents 10 μm. CCT, cytosolic chaperonin–containing tailless complex polypeptide 1; Co-IP, coimmunoprecipitation.

Previously, we have shown that the depletion of Rag GTPases results in the accumulation of defective autolysosomes in germline cells, leading to young egg chamber death under amino acid starvation in *Drosophila* (29). We demonstrated that Rag GTPases are essential for maintaining the low pH in lysosomes, as knockdown of them increases the pH value of autolysosome. Dysregulation of the lysosome formation or regeneration, such as through depletion of Rab7 or Spinster, also leads to lysosome acidification defects, resulting in the accumulation of autolysosome with acidification defects (16, 30, 31, 32). Therefore, we examined the localization of two lysosome proteins Spinster and Rab7. Consistent with our previous finding, depletion of RagA resulted in the accumulation of autolysosomes marked by Atg8a (Fig. 1, *A* and *B*). Interestingly, both Rab7 and Spinster still localized to the autolysosome in *RagA RNAi*, suggesting that there were no obvious defects in lysosome formation and regeneration.

Another possibility for the defective autolysosome accumulation is the v-ATPase assembly defects. The v-ATPase, consisting of multicomponent V0 and V1 domains, pumps protons into the lysosomal lumen to maintain low pH, which is essential for activating lysosomal hydrolases (20). The v-ATPase senses amino acid signaling and interacts with the Rag-LAMTOR complex, which recruits mTORC1 translocation to the lysosome membrane for activation (22). In mammals, mTORC1 regulates lysosomal acidification by controlling the interaction between v-ATPase and the CCT complex (21). When mTORC1 activity is high, the V1 domains reside in the cytosol and associate with the CCT, which inhibits the formation of functional v-ATPase on the lysosome. Inactivation of mTORC1 promotes V1 domains to dissociate from CCT and assemble active v-ATPase. To investigate the interaction between CCT and v-ATPase in *Drosophila*, we cotransfected *Drosophila* S2 cells with the plasmids expressing V5-tagged V1 component Vha36-1 and hemagglutinin (HA)-tagged CCT component CCT1 and then carried out immunoprecipitation (IP) using the V5 antibody. Consistent with the finding in mammalian cells, V5-tagged Vha36-1 coprecipitated HA-tagged CCT1, but not the negative control HA-tagged LacZ (Fig. 1*C*). Given the dynamic regulation of Rag GTPases and mTORC1 on lysosomes (33), we expected that there is physical interaction between Rag GTPases and CCT. Consistent with our hypothesis, the V5-tagged CCT1 coprecipitated by HA-tagged RagA (Fig. 1*D*). These results suggest that Rag GTPases might regulate v-ATPase assembly by associating with the CCT complex.

### Depletion of the CCT attenuates the autolysosome defects in *RagA RNAi*

**Figure 2:**
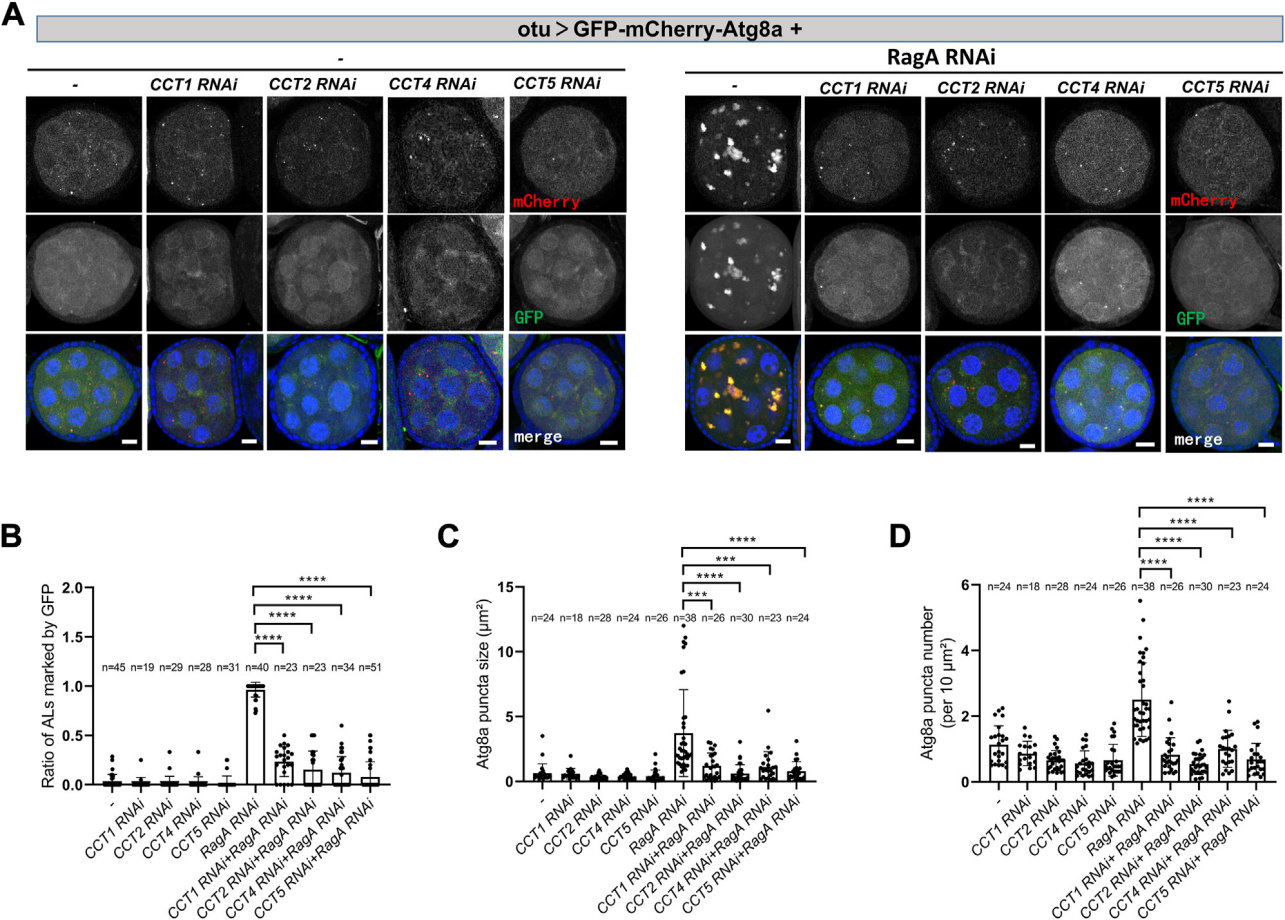
**Depletion of CCT complex attenuates autolysosome accumulation in *RagA RNAi* egg chambers**. *A*, the ovaries were dissected and stained with DAPI. *B*, quantitative analyses of the autolysosomes (ALs, marked by Atg8a-mCherry) positive for GFP. *C*, quantitative analyses of mCherry-Atg8a puncta size. *D*, quantitative analyses of mCherry-Atg8a puncta number (per 10 um^2^). For all graphs, mean and SEM with all data points are shown. n indicates the number of egg chambers. ∗∗*p* < 0.01; ∗∗∗*p* < 0.001; and ∗∗∗∗*p* < 0.0001. The scale bar represents 10 μm. CCT, cytosolic chaperonin–containing tailless complex polypeptide 1.

Given that CCT physically interacts with both Rag GTPases and v-ATPase, we investigate whether depletion of the CCT could rescue the autolysosome accumulation in *RagA RNAi*. When we used the germline-specific driver *otu-nos-GAL4* to knock down the components of the CCT complex, the ovaries were tiny with no egg chamber formed in the ovarioles, suggesting that CCT is essential for GSC activity or cell growth (Fig. S1). We employed the *otu-GAL4*, expressed in germline cells at stage 2 to 8 egg chambers, to knock down CCT (34). The ovaries can grow as normally as the control, suggesting that the CCT complex might affect stem cell development but not cell growth (Fig. S1). To determine autolysosome acidification, we expressed GFP-mCherry-Atg8a in egg chambers using *otu-GAL4*. The fluorescent signals of mCherry, which is resistant to the acid environment, are used to mark autolysosome and the fluorescent signals of GFP, which will be quenched by the acid environment, are used to assess the autolysosome acidification. As previously reported, the fluorescence signal of GFP could not be quenched in *RagA RNAi*, suggesting the autolysosomes were not acidic enough (Fig. 2*A*). Moreover, the size and number of Atg8a-positive puncta were increased in *RagA RNAi*, suggesting the accumulation of autolysosome (Fig. 2, *B–D*). Interestingly, depletion of the CCT complex components CCT1, CCT2, CCT4, or CCT5 diminished the GFP signal and accumulation of autolysosomes in *RagA RNAi* egg chambers (Fig. 2, *A–D*). To further confirm that the knockdown of CCT is enough to rescue the autolysosome degradation defect in *RagA RNAi*, we detected the accumulation of p62, also known as ref (2) P in young egg chambers. Consistently, the accumulation of p62-GFP in *RagA RNAi* is diminished by the depletion of CCT (Fig. S2). It has been reported that the inactivation of mTORC1 breaks the association of CCT with V1-ATPase and promotes v-ATPase assembly in mammalian cells (21). Consistent with this model, the knockdown of mTORC1 activators TOR, Raptor, or Akt rather than the knockdown of mTORC1 inhibitor Tsc1 rescued the accumulation of acidification-deficient autolysosomes in *RagA RNAi* (Fig. S3). Overall, these studies suggest that the defective autolysosome accumulation in *RagA RNAi* results from v-ATPase assembly defects.

### GDP-bound RagA promotes v-ATPase assembly

**Figure 3:**
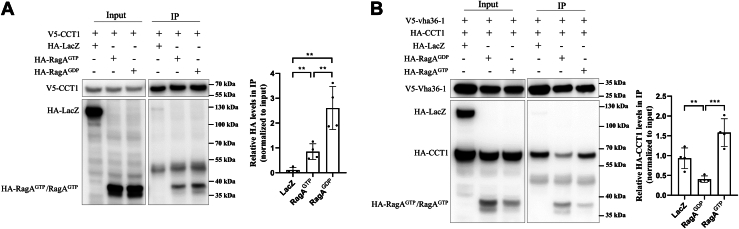
**GDP-bound RagA associates with the CCT complex and regulates v-ATPase assembly**. *A*, S2 cells were cotransfected with V5-tagged CCT1 and HA-tagged RagA^GTP^, RagA^GDP^, or LacZ (control) plasmids. Cells were lysed and immunoprecipitated using beads coated with anti-V5 antibodies. Cell lysates (input) and immunoprecipitates (IP) were detected by Western blot. The experiments were repeated four times, and representative data are shown. *Right*: quantitative analyses of Co-IP of four independent experiments. *B*, S2 cells were cotransfected with V5-tagged Vha36-1 and HA-tagged CCT1 or RagA^GTP^ or RagA^GDP^ or LacZ (control) plasmids. Cells were lysed and immunoprecipitated using beads coated with anti-V5 antibodies. Cell lysates (input) and immunoprecipitates (IP) were detected by Western blot. The experiments were repeated four times, and representative data are shown. *Right*: quantitative analyses of Co-IP of four independent experiments. For all graphs, mean and SEM with all data points are shown. n indicates the number of egg chambers. ∗∗*p* < 0.01; ∗∗∗*p* < 0.001. CCT, cytosolic chaperonin–containing tailless complex polypeptide 1; Co-IP, coimmunoprecipitation; v-ATPase, vacuolar H+-ATPase.

Previously, we have demonstrated that overexpression of GDP-bound RagA (RagA^GDP^) but not GTP-bound RagA (RagA^GTP^) rescued the autolysosome defects in *RagA RNAi* (3, 29). We coexpressed V5-tagged CCT1 and HA-tagged RagA^GTP^ or RagA^GDP^ in *Drosophila* S2 cells, and then carried out IP using the V5 antibody-coated beads to evaluate the interaction between CCT1 and RagA. The amount of RagA^GDP^ pulled down by CCT1 was more than that of RagA^GTP^, suggesting that the interaction between CCT and RagA^GDP^ was stronger (Fig. 3*A*). Next, we determine whether the RagA^GDP^ affects the interaction between CCT and v-ATPase. We coexpressed V5-tagged Vha36-1, HA-tagged CCT1, and RagA^GTP^ or RagA^GDP^ in *Drosophila* S2 cells and performed coimmunoprecipitation using V5 antibody-coated beads. RagA^GDP^ but not RagA^GTP^ tremendously decreased the amount of precipitated HA-tagged CCT1 (Fig. 3*B*). These results indicate that RagA^GDP^ breaks the interaction between CCT1 and v-ATPase V1 domains, promoting v-ATPase assembly.

### Lysosomal localization of Rag GTPases is essential for acidification

**Figure 4:**
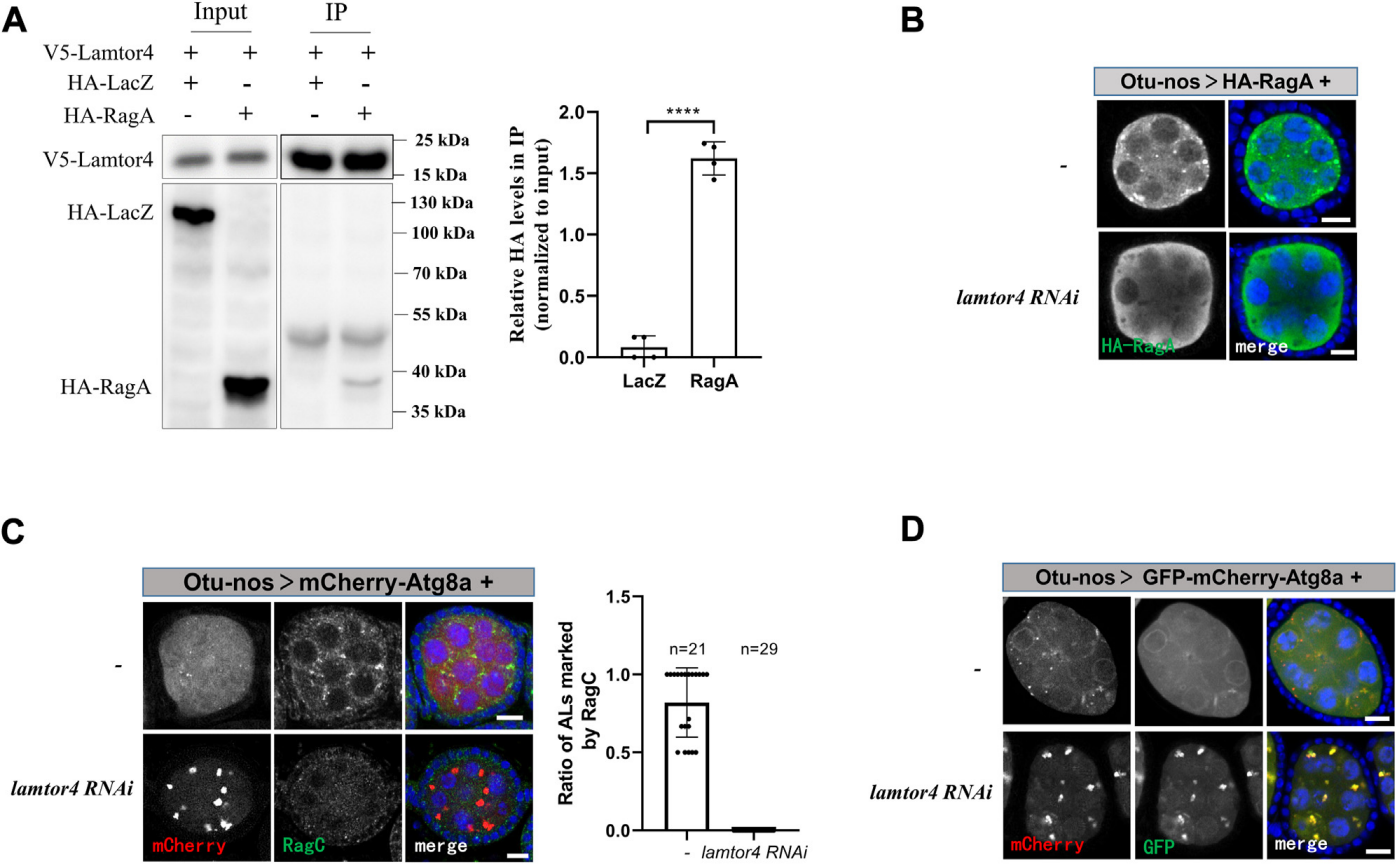
**The localization of Rag GTPases on lysosome is controlled by Lamtor4**. *A*, S2 cells were cotransfected with V5-tagged Lamtor4 and HA-tagged RagA or LacZ (control) plasmids. Cells were lysed and immunoprecipitated using beads coated with anti-V5 antibody. Cell lysates (inputs) and immunoprecipitates (IP) were detected by Western blot. The experiments were repeated four times, and representative data are shown. *Right*: quantitative analyses of Co-IP of four independent experiments. *B*, the ovaries were dissected and stained with anti-HA antibodies and DAPI. *C*, the ovaries were dissected and stained with anti-RagC antibody and DAPI. *Right*: quantitative analyses of the autolysosomes (ALs, marked by Atg8a-mCherry) positive for RagC in the egg chambers. *D*, the ovaries were dissected and stained with DAPI. For all graphs, mean and SEM with all data points are shown. n indicates the number of egg chambers. ∗∗∗∗*p* < 0.0001. The scale bar represents 10 μm. Co-IP, coimmunoprecipitation; LAMTOR, lysosomal adaptor and MAPK and mTOR activator.

In mammals, the LAMTOR complex anchors Rag GTPases to lysosomes (6, 7). To confirm the physical interaction between the LAMTOR complex and Rag GTPases in *Drosophila*, we coexpressed V5-tagged Lamtor4 and HA-tagged RagA in S2 cells and performed IPs using V5 antibody-coated beads. The Lamtor4 specifically coprecipitated with RagA, but not LacZ (Fig. 4*A*). To confirm the localization of Rag GTPases on the lysosome depending on the LAMTOR complex in *Drosophila*, we detected the localization of exogenous HA-tagged RagA in the egg chambers. The HA-RagA positive puncta were in the cytoplasm of the control but were absent in *lamtor4 RNAi* (Fig. 4*B*). To detect the localization of RagC, another Rag GTPases component, we generated an antibody that specifically recognized it (Fig. S4). Similar to RagA, the localization of endogenous RagC on the lysosome, marked by mCherry-Atg8a, was absent in the *lamtor4 RNAi* (Fig. 4*C*). Moreover, knockdown of Lamtor4 resulted in the accumulation of autophagic structures marked by Atg8a (Fig. 4*C*). Next, we expressed GFP-mCherry-Atg8a in egg chambers to determine the lysosomal acidification. Similar to in *RagA RNAi*, the accumulated autolysosomes in *lamtor4 RNAi* had a GFP signal, suggesting that the lysosomal acidification was defective (Fig. 4*D*). These results suggest that the recruitment to the lysosome by the LAMTOR complex is essential for Rag GTPase regulating lysosomal acidification.

### Depletion of the CCT complex attenuates autolysosome defects in *lamtor4 RNAi*

**Figure 5:**
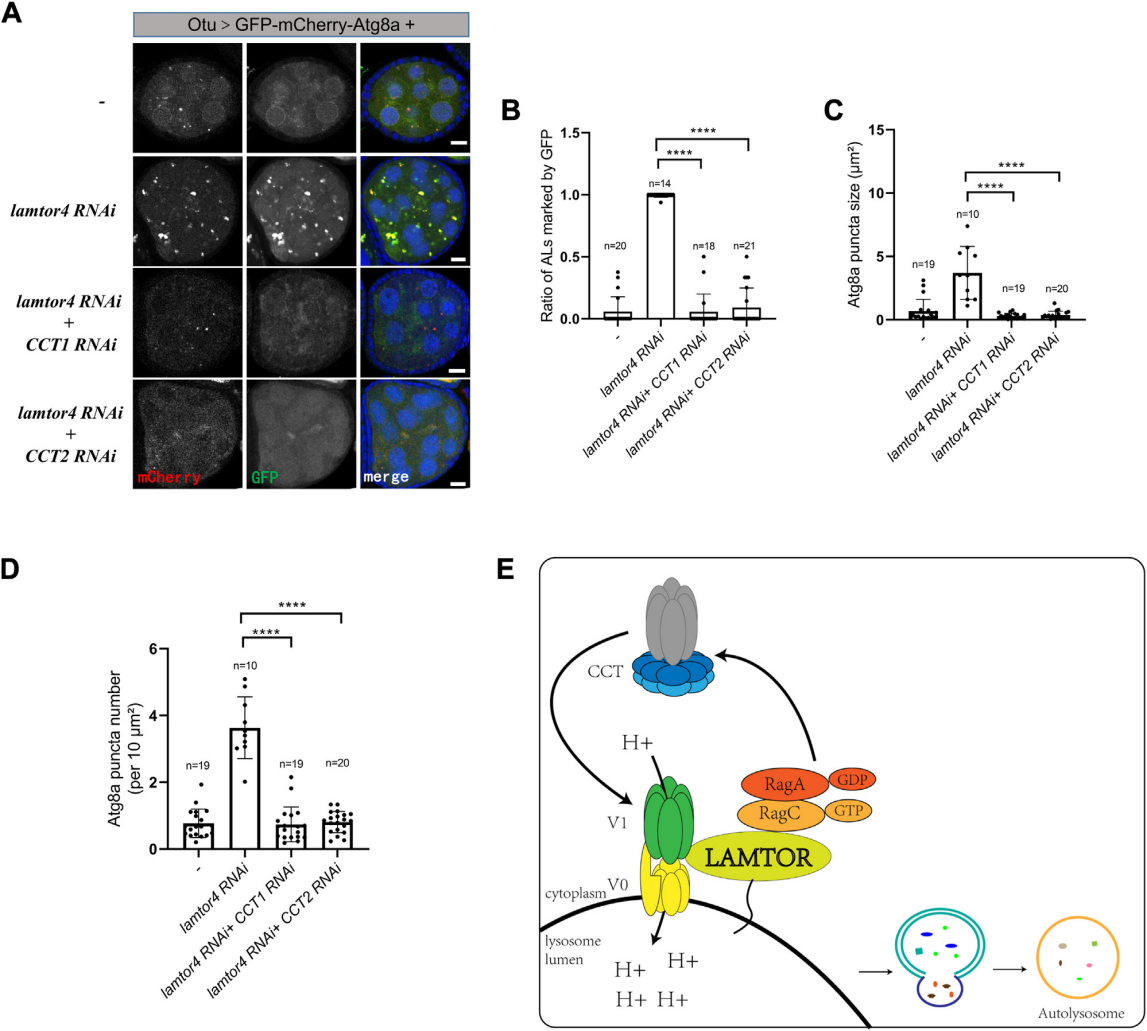
**Depletion of CCT complex attenuates autolysosome accumulation in *lamtor4 RNAi* egg chambers**. *A*, the ovaries were dissected and stained with DAPI. *B*, quantitative analyses of the autolysosomes (ALs, marked by Atg8a-mCherry) positive for GFP. *C*, quantitative analyses of mCherry-Atg8a puncta size. *D*, quantitative analyses of mCherry-Atg8a puncta number (per 10 um^2^). *E*, the model for the RagA^GDP^ controls autolysosome acidification and accumulation by regulating v-ATPase assembly. The RagA^GDP^ breaks the interaction of the CCT complex and v-ATPase V1 domains. The V1 domains translocate from cytosol to lysosome membrane and assemble with V0 domains to form functional v-ATPase, which transports protons cross into the lysosomal to maintain the acidic pH of the lysosomal lumen and degrade contents. For all graphs, mean and SEM with all data points are shown. n indicates the number of egg chambers. ∗∗∗∗*p* < 0.0001. The scale bar represents 10 μm. CCT, cytosolic chaperonin–containing tailless complex polypeptide 1; LAMTOR, lysosomal adaptor and MAPK and mTOR activator; v-ATPase, vacuolar H+-ATPase.

Given the model that Rag GTPases control the lysosomal acidification *via* regulating the interaction between CCT and v-ATPase, we questioned whether the defective v-ATPase assembly underlies autolysosome accumulation in *lamtor4 RNAi*. To address this, we simultaneously knocked down Lamtor4 and CCT components using *otu-Gal4*. Consistently, the autolysosome acidification defects and autolysosome accumulation in *lamtor4 RNAi* were rescued by the knockdown of *CCT1* or *CCT2* (Fig. 5, *A–D*). Interestingly, overexpression of RagA^GDP^ or RagA^wt^ failed to rescue the autolysosome accumulation in the ovaries of *Lamtor 4 RNAi*, where Rag GTPase dissociation from lysosomes (Fig. S5). Taken together, these results suggest that lysosomal localization is essential for Rag GTPases regulating the v-ATPase assembly *via* the CCT complex.

## Discussion

We previously reported that the GDP-bound RagA is essential for autolysosome acidification and degradation (29). In this study, we demonstrated that the lysosomal located GDP-bound RagA breaks the interaction between CCT complex and v-ATPase V1 domains, which promotes the assembly of functional v-ATPase to pump H+ into lysosomal lumen for acidification (Fig. 5*E*).

The lysosomes are degradative and metabolic organelles for macromolecules and damaged organelles degradation in cells (35, 36, 37). The acidic lysosomal lumen, essential for activating hydrolytic enzymes, is maintained by the multisubunit v-ATPase (16). Depletion of RagA induces the accumulation of acidification defective lysosomes in both mammals and *Drosophila* (29, 38, 39). In mammals, it has been suggested that the v-ATPase dysfunction results from the hyperactivation of Mitf in Rag GTPases depletion, which in turn results in the unbalanced expression of the v-ATPase component, breaking the acidic environment in the lumen (40). However, we suggested that the Rag GTPases independently regulate Mitf activity and lysosomal acidification in *Drosophila*, as overexpression of RagA^GTP^ rescued the Mitf activation and RagA^GDP^ rescued the acidification defects in *RagA RNAi* (29). In this study, we further suggest the mechanism of RagA regulates lysosomal acidification: RagA^GDP^ but not RagA^GTP^ breaks the interaction of CCT and v-ATPase V1 domains. The released V1 forms functional v-ATPases with V0 located on the lysosomal membrane and pumps protons into the lysosomal lumen to maintain a low pH value. Interestingly, the RagA^GDP^ is only slightly stronger than RagA^GTP^, suggesting that Rag GTPases regulate lysosomal acidification by breaking the interaction between CCT and V1. Recently it has been reported that inhibition of mTORC1 *via* amino acid starvation or pharmacological inhibitor Torin1 promotes the V1 disassociation with the CCT complex and forms active v-ATPase with the V0 subunit located on the lysosome membrane (21). The affinity of mTORC1 at the lysosome is dynamically regulated *via* cycling the interaction between Rag and LAMTOR complex (33). Under amino acid starvation, the Rag GTPases transform to the inactive state, consisting of RagA^GDP^ and RagC^GTP^, and lose the ability to recruit mTORC1 to the lysosome for activation (3, 4). How GDP-bound RagA and mTORC1 inactivation work together to regulate CCT-V1 complex association under amino acid starvation is an interesting issue for further study.

The v-ATPase takes part in sensing lysosome lumen amino acid levels and controlling mTORC1 activity *via* interacting with the LAMTOR complex (5, 6). The LAMTOR complex—a pentamer containing Lamtor1 to 5 that is anchored on the lysosomal membrane *via* Lamtor1—functions as a scaffold for anchoring Rag GTPases on the lysosome and a GEF toward RagA to activate Rag GTPases, increasing the ability to recruit mTORC1 for activation (5, 22, 41). While the V0 subunit of v-ATPase constitutively associates with the LAMTOR complex, the V1 subunit of v-ATPase reversibly associates with the LAMTOR complex (6, 20, 22). Amino acid stimulation weakens the V1-LAMTOR interaction and promotes the GEF activity of the LAMTOR complex (22). Here, we show that RagA^GDP^ promotes the assembly of v-ATPase *via* breaking in the interaction between CCT and V1, suggesting RagA^GDP^ might regulate the GEF activity of LAMTOR and form a feedback to control mTORC1 activity.

The CCT complex consists of eight subunits and functions as a chaperon (24, 42, 43). Recently it has been reported that the CCT complex promotes cell growth by regulating mTORC1 in *Drosophila* (28). Knockdown of CCT components using the germline driver *otu-nos-Gal4*, expressing from germline stem cell (GSC) to young egg chamber, makes the ovary no germline cytoplasm forming. This result suggests that CCT functions on GSC proliferation and differentiation, which might be beyond mTORC1 inhibition because depletion of TORC1 is not strong enough to cause germline cytoplasm absent. The mechanism of the CCT complex in regulating GSC needs further study.

While the CCT is located in the cytoplasm, Rag GTPases are located on the lysosomal membrane *via* interaction with the LAMTOR complex (5, 21). Depletion of LAMTOR complex component Lamtor4 also results in lysosomal acidification defects, which could be rescued by knockdown of CCT. We think the possibility to explain how Rag GTPases recruit CCT and V1 is that the Rag GTPases recruit CCT-V1 to the lysosome, where the interaction is broken by inactive form Rag GTPases. Depletion of Lamtor4 results in releasing the Rag GTPases to the cytoplasm, which displays a similar effect as depletion of RagA, suggesting the localization of Rag GTPases at the lysosomal membrane is essential for its function. More works are needed to fully understand the mechanism of Rag GTPases regulating v-ATPase assembly.

In conclusion, our work highlights the critical role of GDP-bound RagA in regulating v-ATPase assembly and lysosomal acidification through its interaction with the CCT complex. These findings provide new insights into the molecular mechanisms linking Rag GTPases, v-ATPase, and autophagy.

## Experimental procedures

### *Drosophila* strains

The stocks *RagA RNAi* (THU#1420), *CCT1 RNAi* (THU#1041), *CCT2 RNAi* (THU#3683), *CCT4 RNAi* (THU#1513), *CCT5 RNAi* (TH#201500099.S), *lamtor4 RNAi* (TH#04342.N), *Tor RNAi-1* (THU#201501087.S), and *Tor RNAi-2* (THU#1291) were obtained from Tsinghua Fly Center. The *mCherry RNAi* (BDSC#35785), *UASp-GFP-mCherry-Atg8a* (BDSC#37749), *UASp-mCherry-Atg8a* (BDSC#37750), and *UAS-spinster-myc-GFP* (BDSC#39668), and *Tsc1 RNAi* (BDSC#35144) were obtained from Bloomington *Drosophila* Stock Center. The *otu-GAL4*, *otu-nos-GAL4*, *UASp-RagA*^*wt*^, *UASp-RagA*^*GTP*^, and *UASp-RagA*^*GDP*^ were described previously (29). All fly stocks were maintained on BDSC standard cornmeal medium.

### Immunofluorescence

The *Drosophila* ovaries were dissected in phosphate buffer and fixed in 4% paraformaldehyde for 20 min. Then the ovaries were washed four times with 0.5% Triton X-100 PBS (0.5% PBST) for 20 min. The ovaries were incubated for 6 h at room temperature in primary antibodies. The antibodies were diluted in PBST: mouse anti-HA at 1:500 (Santa Cruz Biotechnology, #Sc-7392), mouse anti-Rab7 at 1:100 (DSHB, #AB_2722471), and rabbit anti-V5 at 1:5000 (Proteintech, #14440-1-AP). Subsequently, the ovaries were washed four times for 20 min in PBST and then incubated 2 h in secondary antibodies (Alexa Fluor 488 or Alexa Fluor 594 at 1:2000). The ovaries were washed 4 times with PBST for 20 min and then mount ovaries using fluoroshield media containing 4′,6-diamidino-2-phenylindole (DAPI), a DNA-specific fluorescent stain (Sigma-Aldrich, #F6057). We use a Zeiss 880 confocal microscope to access fluorescent images. The genotypes of *Drosophila* used for analysis are listed in Table S1.

### Plasmid construction

The plasmids were constructed using the homologous recombination kit according to the manufacturer’s instructions (Vazyme, #C112-01). The pAC5.1-His-V5 vector (Invitrogen, #V411020) was linearized with restriction enzyme KpnI and XhoI, while pAC5.1-HA was linearized by EcoRI and XbaI. Gene fragments encoding CCT1, vha36-1, lamtor4, RagA, RagA-T16N, and RagA-Q61L were PCR-amplified and gel purified. Gene fragments of CCT1, vha36-1, and lamtor4 were inserted into pAC5.1A-V5 to construct a series of plasmids. Gene fragments of RagA, RagA-T16N, RagA-Q61L, and CCT1 were inserted into pAC5.1A-HA to construct a series of plasmids for cell transfection. The coding region of RagC was amplified using specific primers and inserted into the pET28a vector to generate the pET28a-RagC antigen plasmid. The PCR primers used are listed in Table S2.

### Generation of mouse polyclonal RagC antibody

Transform the pET28a-RagC recombinant plasmid into *Escherichiacoli* strain BL21 for further cultivation. Use 0.5 mM concentration of IPTG (Sangon Biotech B541007; #367-93-1) to induce the expression of RagC antigen. The cells were lysed by ultrasonic fragmentation. Recombinant RagC antigen was purified using a His-tagged protein purification kit (CoWin Biotech, #CW0894) following the manufacturer’s instructions. The mouse polyclonal anti-RagC antibodies were generated by the Beijing Protein Innovation company.

### Cell culture and cell transfection

*Drosophila* S2 cells were cultured in Schneider’s *Drosophila* Media (Merck, #S9895), which was supplemented with 10% fetal bovine serum (Thermo Fisher Scientific, #A3160802). Transient transfection was executed using the Lipomaster 3000 transfection reagent according to the manufacturer’s instruction (Vazyme, TL301-01). Cells were harvested 48 h after transfection for further detection.

### IP and Western blotting

The S2 cells transfected with specific plasmids were dissected in Radioimmunoprecipitation Assay buffer (Merck, #20-188) containing complete protease inhibitors (Roche, #1697498001) and phosphatase inhibitors (Roche, #4906845001). The Cell lysates were incubated with 20 μl HA or V5 antibody-coated magnet beads for 6 h at room temperature. The beads were collected and washed eight times with lysis buffer and then detected by Western blot. The antibodies were used at the following concentrations for Western blot: rabbit anti-HA at 1:2000 (Beyotime, #AF0039) and rabbit anti-V5 at 1:5000 (Invitrogen, #R960-25). For all Western blot image analyses, pixel values of a single lane were normalized to the average value of all lanes. The IPs were normalized to the inputs indicated in the respective immunoblot figure.

### Statistical analyses

Statistical analysis was performed using GraphPad Prism 8 (https://www.graphpad.com/). A two-tailed Student’s *t* test was used to determine the significance of the difference between samples. Statistical significance is shown by stars: ∗, *p* < 0.05; ∗∗, *p* < 0.01; ∗∗∗, *p* < 0.001; and ∗∗∗∗, *p* < 0.0001; NS, no significant. For all graphs, mean and SEM with all data points are shown.

## Data availability

All data are contained within the article and the supporting information.

## Supporting information

This article contains supporting information.

## Conflict of interest

The authors declare that they have no conflicts of interest with the contents of this article.
