# Supplementary material for: Rag GTPases control lysosomal acidification by regulating v-ATPase assembly in Drosophila
Source: J Biol Chem. 2025 Jun 19;301(7):110400. doi: 10.1016/j.jbc.2025.110400 (PMC12332402; doi:10.1016/j.jbc.2025.110400)
Supplement: Supplementary Material [file mmc1.pdf]

Rag GTPases control lysosomal acidification by regulating v-ATPase assembly in *Drosophila*

Ying Zhou[1, 2], Xiaodie Yang[1, 2], Wenyu Xu[1, 2], Sulin Shen[1, 2], Weikang Fan[1, 2], Guoqiang Meng[1,2],

Yang Cheng[1, 2], Yingying Lu[1, 2], Youheng Wei[1, 2, 3,#]

Supporting information

Supporting figures:

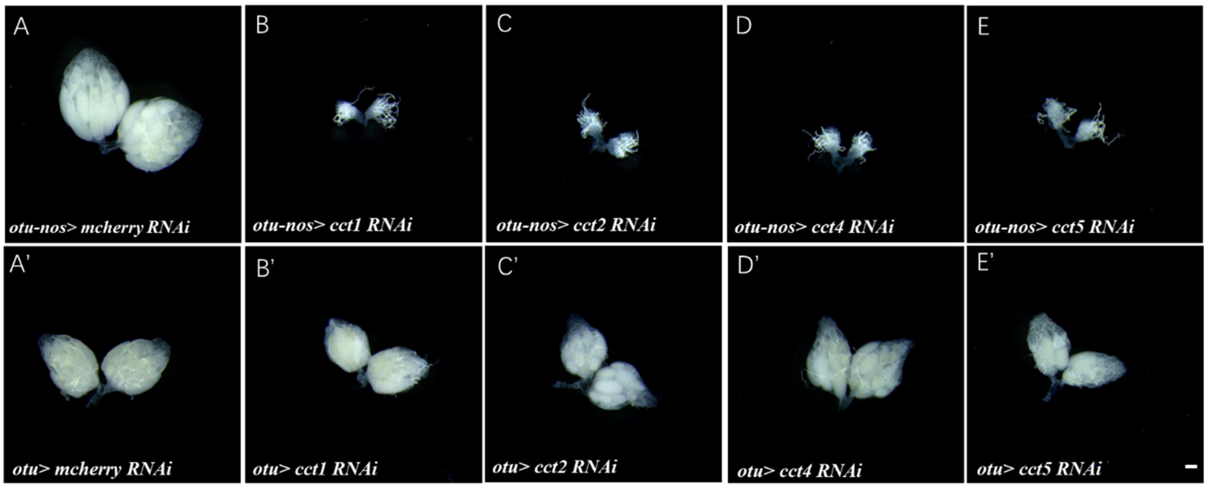

Figure S1. CCT complex affects *Drosophila* ovary growth. The ovaries from (A) *otu-nos-GAL4;;UAS-mCherry RNAi,* (B) *otu-nos-GAL4;;UAS-CCT1 RNAi,* (C) *otu-nos-GAL4;;UAS-CCT2 RNAi,* (D) *otu-nos-GAL4;;UAS-CCT4 RNAi,* (E) *otu-nos-GAL4; UAS- CCT5 RNAi,* (A') *otu-GAL4;;UAS-mCherry RNAi,* (B') *otu-GAL4;;UAS-CCT1 RNAi,* (C') *otu-GAL4;;UAS-CCT2 RNAi,* (D') *otu-GAL4;;UAS-CCT4 RNAi,* and (E') *otu-GAL4;UAS-CCT5 RNAi*. Scale bar: 100μm.

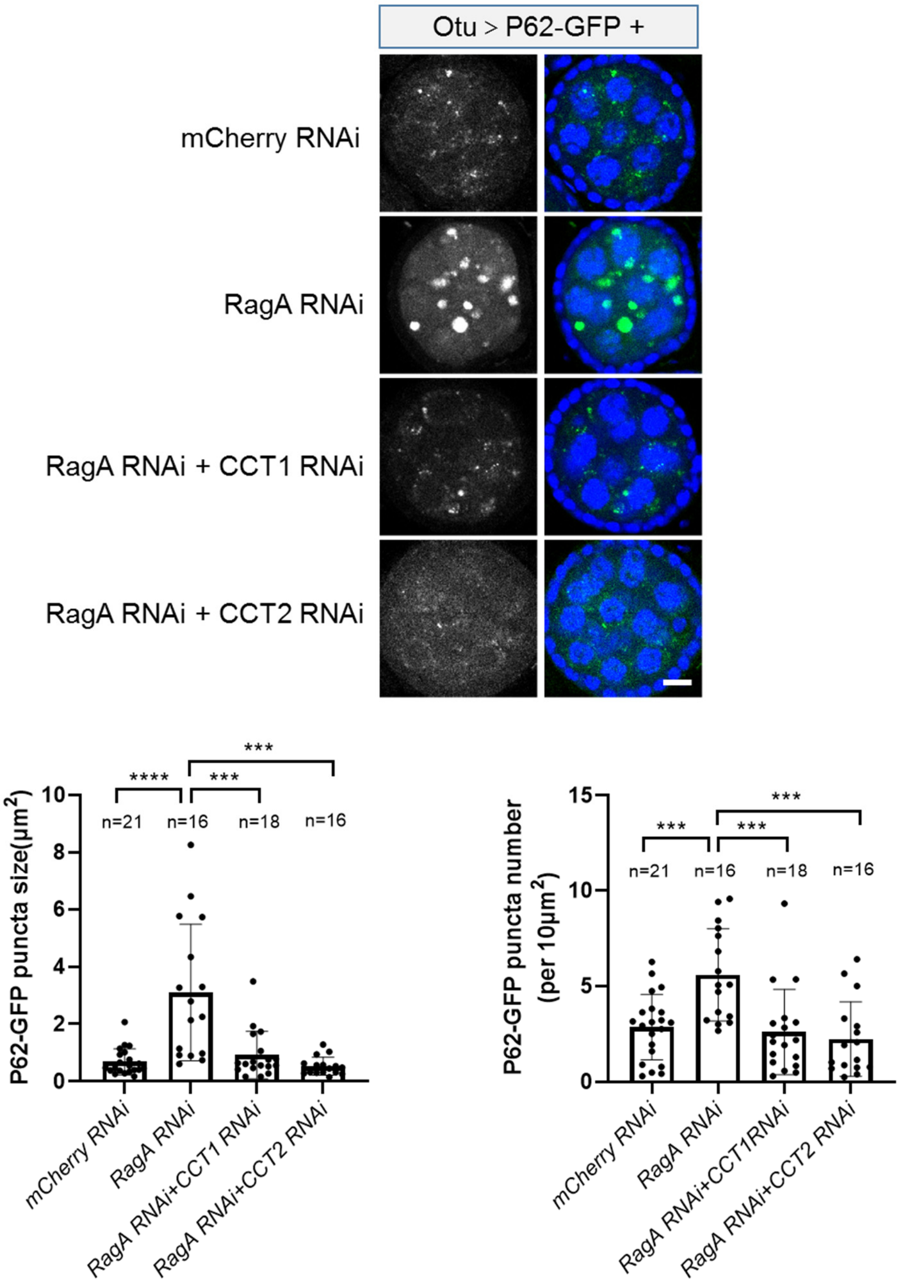

Figure S2. Knockdown of CCT promotes autolysosomal degradation in *RagA RNAi.* The ovaries from *otu-GAL4;UASp-P62-GFP;UAS-mCherry RNAi*, *otu-GAL4;UASp-P62-GFP;UAS-RagA RNAi*, *otu-GAL4;UASp-P62-GFP;UAS-RagA* RNAi/*UAS-CCT1 RNAi* and *otu-GAL4;UASp-P62-GFP;UAS-RagA RNAi*/*UAS-CCT2 RNAi* flies were fixed and stained by DAPI. Down, quantitative analyses of P62-GFP puncta size and P62-GFP puncta number (per 10 um^2). For all graphs, mean and SEM with all data points are shown. n indicates the number of egg chambers. ***p < 0.001, ****p < 0.0001. Scale bar: 10 μm.

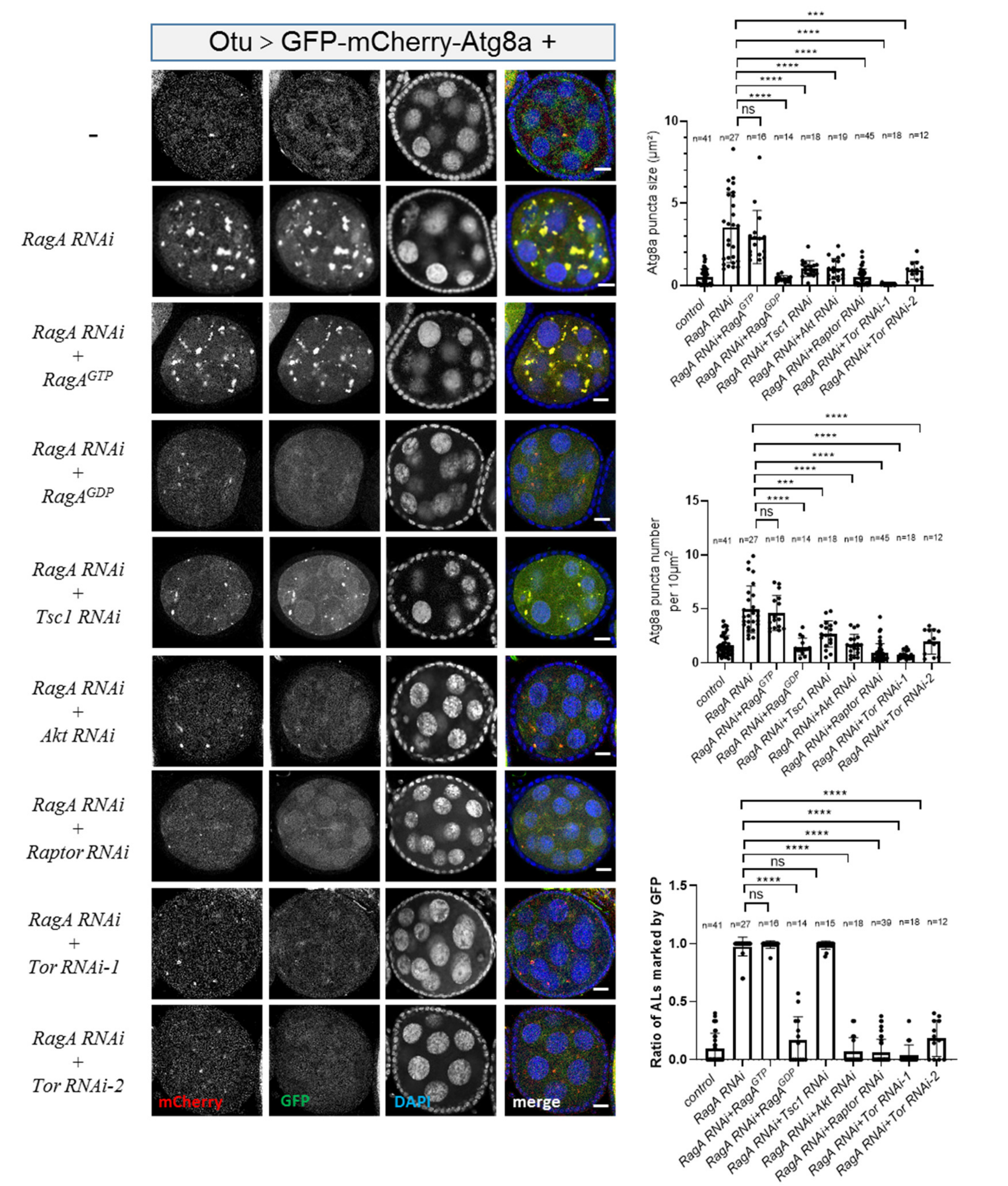

Figure S3. Inactivation of mTORC1 promotes autolysosomal degradation in *RagA RNAi*. The ovaries from *otu-GAL4;UASp-GFP-mCherry-Atg8a*, *otu-GAL4;UASp-GFP-mCherry-Atg8a;UAS-RagA RNAi*, *otu-GAL4;UASp-GFP-mCherry-Atg8a/UASp-RagA^{GTP};UAS-RagA RNAi*, *otu-GAL4;UASp-GFP-mCherry-Atg8a/UASp-RagA^{GDP};UAS-RagA RNAi*, *otu-GAL4;UASp-GFP-mCherry-Atg8a;UAS-RagA RNAi/UAS-Tsc1 RNAi*, *otu-GAL4;UASp-GFP-mCherry-Atg8a;UAS-RagA RNAi/UAS-Akt RNAi*, *otu-GAL4;UASp-GFP-mCherry-Atg8a/UAS-Raptor RNAi;UAS-RagA RNAi*, *otu-GAL4;UASp-GFP-mCherry-Atg8a/UAS-Tor RNAi-1;UAS-RagA RNAi*, and *otu-GAL4;UASp-GFP-mCherry-Atg8a;UAS-RagA RNAi/UAS-Tor RNAi-2* were fixed and stained by DAPI. Right, Quantitative analyses of mCherry-Atg8a puncta size, mCherry-Atg8a puncta number (per 10 um^2), and the autolysosomes (ALs, marked by Atg8a-mCherry) positive for GFP. For all graphs, mean and SEM with all data points are shown. n indicates the number of egg chambers. ***p < 0.001, ****p < 0.0001. Scale bar: 10 μm.

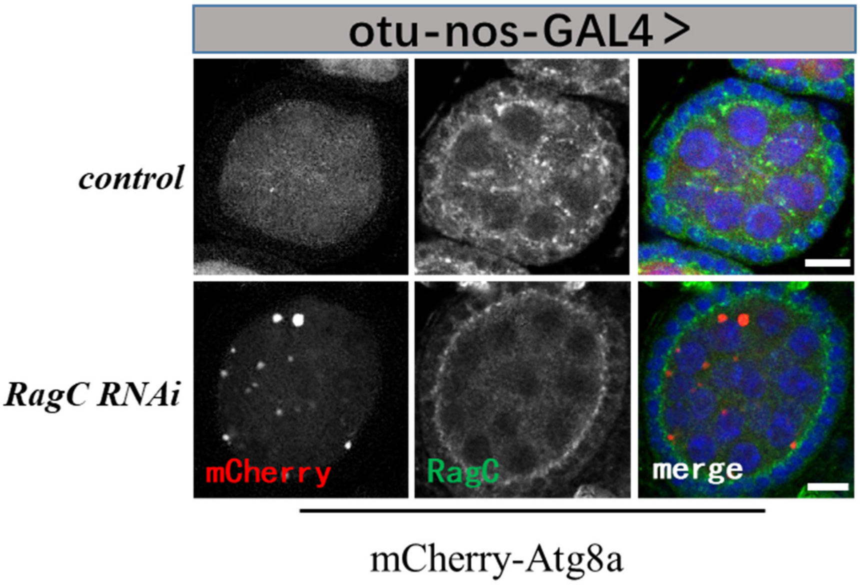

Figure S4. The RagC antibody works well in immunofluorescent. The ovaries from *otu-nos-GAL4;UASp-mCherry-Atg8a* (control) and *otu-nos-GAL4; UASp-mCherry-Atg8a/RagC RNAi* flies were fixed and stained by RagC antibody. Scale bar: 10 μm.

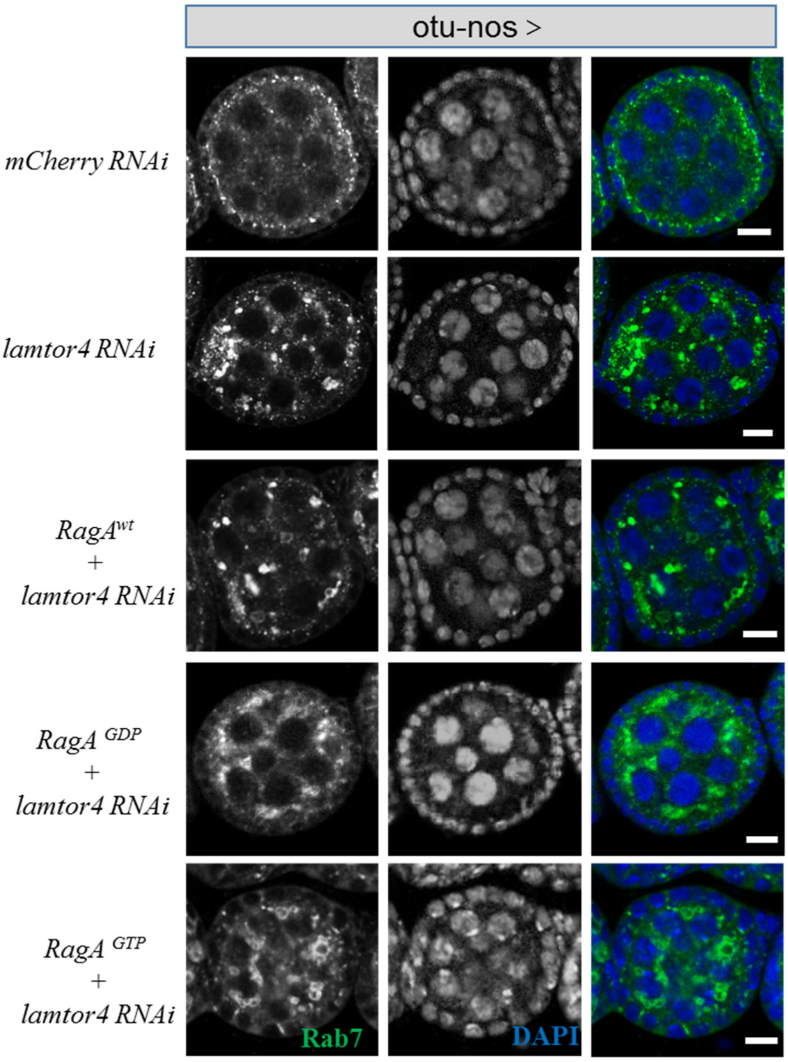

Figure S5. Overexpression of RagA could not rescue the autolysosome accumulation in *lamtor4 RNAi*. The ovaries from *otu-GAL4;UAS-mCherry RNAi*, *otu-GAL4;UAS-lamtor4 RNAi*, *otu-GAL4;UAS-lamtor4 RNAi/UASp-RagA*^{wt}, *otu-GAL4;UAS-lamtor4 RNAi/UASp-RagA*^{GDP}, and *otu-GAL4;UAS-lamtor4 RNAi/UASp-RagA*^{GTP} were fixed and stained by anti-Rab7 and DAPI. Scale bar: 10 μm.

Supporting tables:

Supplemental Table 1. Genotypes in each figure

	Genotypes
**Figure 1A**	*otu-nos GAL4; UASp-mCherry-Atg8a*<br>*otu-nos- GAL4; UASp-mCherry-Atg8a; UAS-RagA RNAi*
**Figure 1B**	*otu-nos GAL4; UASp-mCherry-Atg8a; UAS-Spinster-GFP*<br>*otu-nos- GAL4; UASp-mCherry-Atg8a; UAS-Spinster-GFP/ UAS-RagA RNAi*
**Figure 2A-2D**	*otu-GAL4; UASp-GFP-mCherry-Atg8a*<br>*otu-GAL4; UASp-GFP-mCherry-Atg8a; UAS-CCT1 RNAi*<br>*otu-GAL4; UASp-GFP-mCherry-Atg8a; UAS-CCT2 RNAi*<br>*otu-GAL4; UASp-GFP-mCherry-Atg8a; UAS-CCT4 RNAi*<br>*otu-GAL4; UASp-GFP-mCherry-Atg8a / UAS-CCT5 RNAi*<br>*otu-GAL4; UASp-GFP-mCherry-Atg8a; UAS-RagA RNAi*<br>*otu-GAL4; UASp-GFP-mCherry-Atg8a; UAS-CCT1 RNAi/ UAS-RagA RNAi*<br>*otu-GAL4; UASp-GFP-mCherry-Atg8a; UAS-CCT2 RNAi/ UAS-RagA RNAi*<br>*otu-GAL4; UASp-GFP-mCherry-Atg8a; UAS-CCT4 RNAi/ UAS-RagA RNAi*<br>*otu-GAL4; UASp-GFP-mCherry-Atg8a/ UAS-CCT5 RNAi; UAS-RagA RNAi*
**Figure 4B**	*otu-nos-gal4; UASp-HA-$RagA^{WT}$*<br>*otu-nos-gal4; UASp-HA-$RagA^{WT}$/ UAS-lamtor4 RNAi*
**Figure 4C**	*otu-nos-gal4; UASp-mCherry-Atg8a*<br>*otu-nos-gal4; UASp-mCherry-Atg8a / UAS-lamtor4 RNAi*
**Figure 4D**	*otu-nos-gal4; UASp-GFP-mCherry-Atg8a*<br>*otu-nos-gal4; UASp-GFP-mCherry-Atg8a / UAS-lamtor4 RNAi*
**Figure 5A-5D**	*otu-GAL4; UASp-GFP-mCherry-Atg8a*<br>*otu-GAL4; UASp-GFP-mCherry-Atg8a/ UAS-lamtor4 RNAi*<br>*otu-GAL4; UASp-GFP-mCherry-Atg8a/ UAS-lamtor4 RNAi; UAS-CCT1 RNAi*<br>*otu-GAL4; UASp-GFP-mCherry-Atg8a/ UAS- lamtor4 RNAi; UAS- CCT2 RNAi*

Table S2. Primers used for generating plasmids

Primer	Sequence
CCT1 Forward	ACTAGTCCAGTGTGGTGGAATTCatgtcgaccctggcctctcc
CCT1 Reverse	GGAACATCGTATGGGTATCTAGAaccgtccagctcgccggcag
RagA Forward	ATCCAGAGACCCCGGATCGGGGTACCatgaagaaaaaggtgttac
RagA Reverse	AACATCGTATGGGTATCTAGACTCGAGatggtacctttggccatg
RagA-Q61L Forward	actgtggcgg tctggagggc ttc
RagA-Q61L Reverse	gaagccctccagaccgccacagt
RagA-T16N Forward	ccggaaagaaca gcatgcgctc
RagA-T16N Reverse	gagcgcatgctgttctttccgg
vha36-1 Forward	TCCAGAGACCCCGGATCGGGGTACCatgtccggaaaagataggc
vha36-1 Reverse	TTCGAAGGGCCCTCTAGACTCGAGgaacagcacgtcgtcatcg
CCT1 Forward	TCCAGAGACCCCGGATCGGGGTACCatgtcgaccctggcctctcc
CCT1 Reverse	CTTCGAAGGGCCCTCTAGACTCGAGaccgtccagctcgccggcag
lamtor4 Forward	TCCAGAGACCCCGGATCGGGGTACCatgttgaaaatggacagggaa
lamtor4 Reverse	CTTCGAAGGGCCCTCTAGACTCGAGagccagcactgccccggaat
